# Increasing anaphylaxis events in Western Australia identified using four linked administrative datasets^∗^

**DOI:** 10.1016/j.waojou.2020.100480

**Published:** 2020-11-13

**Authors:** Sandra M. Salter, Ross J. Marriott, Kevin Murray, Samantha L. Stiles, Paul Bailey, Raymond J. Mullins, Frank M. Sanfilippo

**Affiliations:** a School of Allied Health, Faculty of Health and Medical Sciences, The University of Western Australia, M315, 35 Stirling Highway, Perth, WA, 6009, Australia; b School of Population and Global Health, Faculty of Health and Medical Sciences, The University of Western Australia, Perth, WA, 6009, Australia; c St John WA, Belmont, Western Australia, 6104, Australia; d Suite 1, John James Medical Centre, 175 Strickland Crescent, Deakin, ACT, 2600, Australia; e School of Population and Global Health, The University of Western Australia, Perth, Western Australia, 6009, Australia

**Keywords:** Anaphylaxis, Epidemiology, Linked data, Events, Australia, EDDC, Emergency Department Data Collection, EPAWA, Epidemiology of Anaphylaxis in Western Australia, HMDC, Hospital Morbidity Data Collection, ICD-9-CM, International Classification of Diseases, 9th revision, Clinical Modification, ICD-10-AM, International Statistical Classification of Diseases and Related Health Problems, 10th revision, Australian Modification, IRSAD, Index of Relative Socio-Economic Advantage and Disadvantage, SEIFA, Socio-Economic Indexes for Areas, WAAC, Western Australian Anaphylaxis Cohort, WADLS, Western Australian Data Linkage System

## Abstract

**Background:**

Anaphylaxis events are increasing worldwide, based on studies of single administrative datasets including hospital admissions, emergency room presentations, and prescription and medical claims data. Linking multiple administrative datasets may provide better epidemiological estimates, by capturing a greater number of anaphylaxis events occurring at the individual level. In this linked data study in Western Australia, we combined 4 population-based datasets to identify anaphylaxis events, factors influencing occurrence, and change in event rates from 2002 to 2013.

**Methods:**

Four linked administrative datasets from the Western Australian Data Linkage System were used, representing ambulance attendances, emergency department presentations, hospital inpatient admissions and death registrations. An anaphylaxis cohort was identified using ICD-9-CM, ICD-10-AM and additional anaphylaxis diagnosis codes, with event rates calculated. We explored the impact of age, gender, cause, Indigenous status and socioeconomic index on event rates. Standard Poisson regression models were used to examine the significance of the change in anaphylaxis event rates over time.

**Results:**

A total 12,637 individuals (mean age 31.8 years, 49.6% female) experienced 15,462 anaphylaxis events between 2002 and 2013 (97.5% in non-Indigenous patients and 59.5% residing in the area of greatest socioeconomic advantage). Anaphylaxis event rates increased from 15.4 to 82.5/10^5^ population between 2002 and 2013. The greatest increase in anaphylaxis events was seen in those coded as unspecified anaphylaxis (all ages, males and females combined, p < 0.001), with the highest rates of unspecified anaphylaxis in males 0–4 years (171.9/10^5^ population in 2013), and females 15–19 years (104.0/10^5^ in 2013). The average annual percent increase (95% CI) for food-related anaphylaxis was 9.2% (6.6–12.0); for medication-related anaphylaxis was 5.8% (4.5–7.1); and for unspecified anaphylaxis was 10.4% (9.8–11.0); all p < 0.001. There was a significant increase in ambulance attendance, emergency presentations and inpatient admissions for anaphylaxis between 2002 and 2013, with emergency presentations (56.0/10^5^ population), inpatient admissions (43.2/10^5^), and ambulance attendance (21.6/10^5^) highest in 2013. Only 25 anaphylaxis-related deaths were recorded in the mortality register with no significant change in rates over time.

**Conclusion:**

Using multiple linked administrative datasets, we identified significantly higher rates of total anaphylaxis than previously reported, with more than 5-fold increases in anaphylaxis events between 2002 and 2013. While the combination of 4 population-level datasets provides a more comprehensive capture of cases, even at the individual dataset level, admission rates for anaphylaxis in Western Australia are substantially higher than those previously reported for similar time periods, both in Australia and worldwide.

## Introduction

Anaphylaxis is defined as a serious allergic reaction that is rapid in onset and might cause death.^1^ The public health burden of anaphylaxis is considered to be increasing worldwide based on studies of single administrative datasets which provide deidentified, population-level health data, such as that from hospital admissions, emergency room presentations, prescription and insurance data.^2^, ^3^, ^4^, ^5^, ^6^, ^7^, ^8^, ^9^, ^10^ Interpretation of such data, however, is subject to a number of caveats: (a) the potential for underestimating the burden of anaphylaxis (due to differences in health seeking behaviour, those cared for in the community or who never seek medical attention), (b) the potential for overestimation (due to an inability to distinguish between single and multiple episodes in the same patient), and (c) the potential for variability in diagnostic accuracy.

Linking multiple administrative datasets has the potential to circumvent many of these issues, by capturing a larger number of unique anaphylaxis events across multiple levels of the health system, enabling researchers to identify which records from multiple datasets belong to the same person and event, and thereby provide the most comprehensive epidemiological estimates.^11^

The Epidemiology of Anaphylaxis in Western Australia (EPAWA) study is a multi-phase linked data study that combined population-level ambulance, emergency department, hospital inpatient admissions, and mortality datasets to identify the Western Australian Anaphylaxis Cohort (WAAC) and determine the epidemiology of anaphylaxis in Western Australia. Here we report phase 1, with objectives to: (i) identify the population representing all anaphylaxis individuals in Western Australia for the period 1980–2014 (the WAAC); (ii) determine annual event rates of anaphylaxis for the common time period across all datasets (2002–2013), in a subset of the WAAC; and (iii) explore the impact of age, gender, cause and dataset source on anaphylaxis event rates, and determine change in these event rates including fold change and annual percentage change over time, in the WAAC subset.

## Methods

### Data source

The Western Australian Data Linkage System (WADLS) is a system of person-specific linkages within and between health and non-health datasets, maintained by the Western Australian Department of Health. The data system includes records from 1969 onwards, and contains over 150 million records spanning more than 50 routinely linked datasets for the population of Western Australia.^12^ Data linkage is carried out using demographic information such as name, date of birth and address, and uses probabilistic matching with manual review where links are uncertain, to ensure the highest quality of linked data.^11^

The EPAWA study utilised 4 datasets relevant to anaphylaxis, for which data were available for different time periods: the Hospital Morbidity Data Collection (commonly known as inpatient admissions; HMDC; 1980–2015), the Emergency Department Data Collection (EDDC; 2002–2015), Western Australian Ambulance records (Ambulance; 1995–2013) and Western Australian Death Registrations (Deaths; 1981–2016). In order to examine the changing epidemiology of anaphylaxis, it was essential to first establish the historical Western Australian anaphylaxis population using as broad a time period as possible. In this paper (phase 1 of EPAWA), we report the identification of the historical population and then examine anaphylaxis events in a subset cohort over a suitable time period.

### Study population

#### Identification of the Western Australian anaphylaxis cohort (WAAC)

Individuals with anaphylaxis recorded in any of the HMDC, EDDC, Ambulance or Deaths datasets for the time period 1980–2014 comprised the Western Australian Anaphylaxis Cohort (WAAC). Anaphylaxis individuals were identified in each dataset separately using International Classification of Diseases (ICD) anaphylaxis diagnosis codes for ICD-9-CM (Clinical Modification): 995.0 (anaphylactic shock; includes anaphylactic reaction, anaphylaxis not otherwise specified, anaphylactic reaction due to adverse event of correct medicinal substance properly administered), 995.60–995.69 (anaphylactic shock due to adverse food reaction/s), 999.4 (anaphylactic reaction to serum) and ICD-10-AM (Australian Modification): T78.0 (anaphylactic shock due to adverse food reaction), T78.2 (anaphylactic shock not otherwise specified), T80.5 (anaphylactic shock due to serum), T88.6 (anaphylactic shock due to adverse event of correct drug or medicament properly administered), plus dataset-specific anaphylaxis codes (local codes in EDDC and Ambulance datasets). The Ambulance dataset in particular does not utilise ICD-codes, and as such no anaphylaxis cause is attributed in coding. Therefore all anaphylaxis cases from Ambulance are included in this report as unspecified anaphylaxis.

**Fig. 1:**
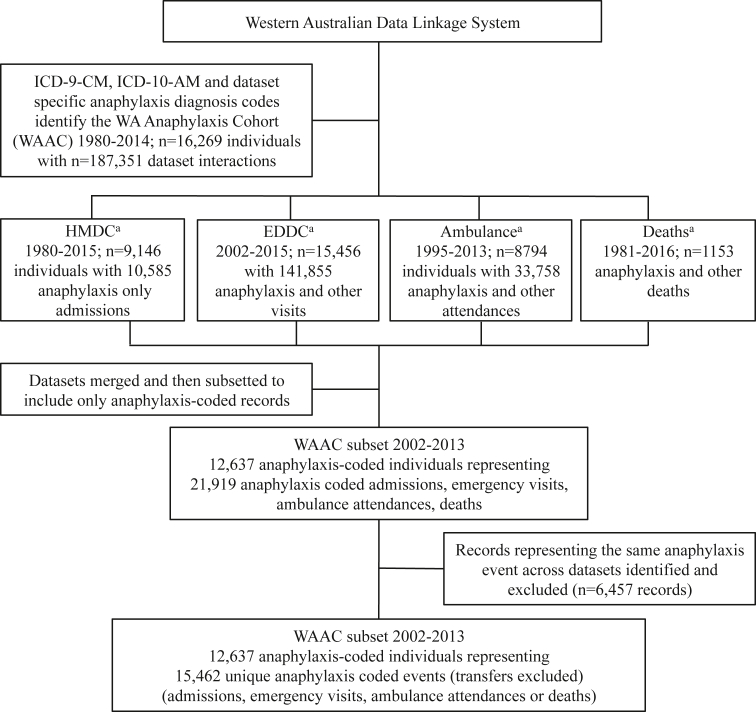
**Identification of the Western Australian Anaphylaxis Cohort,** ICD: International Classification of Diseases, CM: Clinical Modification, AM: Australian Modification, WAAC: Western Australian Anaphylaxis Cohort, HMDC: Hospital Morbidity Data Collection, EDDC: Emergency Department Data Collection, ^a^ Data for HMDC and EDDC were extracted to end 2015; Ambulance to end 2013; Deaths to end 2016, in accordance with data available at the time

Linked records (all anaphylaxis-coded records for each individual in the WAAC, plus all other non-anaphylaxis records for those individuals) were extracted in accordance with the time periods available (as above), for EDDC (n = 141,855), Ambulance (n = 33,758) and Deaths (n = 1153). For HMDC, only anaphylaxis-coded records were extracted (n = 10,585). Collectively these records represent the combined health encounters of the WAAC (n = 16,269 individuals for the period 1980–2014 with a total 187,351 health records for the period 1980 up to 2016); Fig. 1.

### Study cohort

#### Anaphylaxis events in the WAAC subset, 2002–2013

The common time period for which all datasets were available was 2002–2013. Therefore, the most comprehensive anaphylaxis estimates from the WAAC will exist within this time period. Anaphylaxis records from this time period were identified separately in each dataset using the ICD anaphylaxis diagnosis codes above, and Ambulance-specific anaphylaxis problem codes. We searched symptom and principal diagnosis fields in datasets from the EDDC, principal and secondary diagnoses fields in HMDC, and all listed cause of death codes in Deaths. Text fields in Ambulance and Deaths were also searched for the “anaph” substring. The 4 datasets were merged, with only anaphylaxis records included in analysis (n = 12,637 individuals representing 15,462 anaphylaxis events for the period 2002–2013); Fig. 1.

Individuals across this time period may have experienced an anaphylaxis event that resulted in a transfer from one part of the health system to another. In order to prevent double counting, records pertaining to the same anaphylaxis event existing in more than 1 dataset (eg, attendance by ambulance then visit to emergency; emergency to hospital; transfer from one hospital to another; death after interaction with ambulance, emergency or inpatients), were identified as transfer duplicates, and removed. In this case, the first record in the sequence was retained as that identifying the event. Please see the Supplemental Appendix for explanation of identification of transfer records and removal of duplicates.

We considered anaphylaxis events against key variables gender, age, anaphylaxis cause type, Indigenous status, and the Socio-Economic Indexes for Areas (SEIFA). The SEIFA used was the Index of Relative Socio-Economic Advantage and Disadvantage (IRSAD), which is a general measure of peoples’ access to material and social resources, and their ability to participate in society. This index ranks areas on a continuum from most disadvantaged to most advantaged and represents the collective socio-economic characteristics of the people living in an area.^13^ The IRSAD scores from the 2011 Australian census were used, grouped into quintiles for analysis, with quintile 1 representing the most disadvantaged area and quintile 5 representing the least disadvantaged area.

Where possible, missing values for variables (such as age or gender) in any dataset were obtained from values existing in the other linked datasets. Where the patient only had an age or gender record in 1 dataset and the missing values could not be obtained, the record was excluded from analysis (representing a total of 40 events in this study).

### Analysis

Demographic variables (gender, age, Indigenous status, and SEIFA) were described as mean and standard deviation (SD), or count and percentage for all years 2002–2013. Annual anaphylaxis event rates (expressed as the number of anaphylaxis events per 10^5^ population, using the merged linked datasets) were calculated according to the formula below, where group is the gender and/or age group of interest, and cause type is the ICD-coded causes: food, medication and unspecified (respectively T78.0, T88.6, T78.2, or equivalent as previously stated):

$$Event\;Rate\;{\left(per\;10^{5}\;population\right)}_{\left[cause\;type,\;group,\;year\right]}\;=\;\frac{Count_{\left[cause\;type,\;group,\;year\right]}}{Population_{\left[group,\;year\right]}}\times 100,000$$

The denominator (Population) was the estimated resident population of Western Australia for each year^14^ for the corresponding demographic grouping (group) in the numerator, as a measure of anaphylaxis occurrence for each year.

We also examined anaphylaxis to serum (T80.5 or equivalent), however this was found to be negligible in our event estimates and is not included in this report.

Dataset specific anaphylaxis rates (expressed as the number of anaphylaxis events per 10^5^ population for each individual dataset) were calculated according to the formula below:

$$Event\;Rate\;{\left(per\;10^{5}\;population\right)}_{\left[cause\;type,\;dataset,\;year\right]}\;=\;\frac{Count_{\left[cause\;type,\;dataset,\;year\right]}}{Population_{\left[year\right]}}\times 100,000$$

Dataset-specific event rates are stand-alone for each separate dataset; thus, transfers were not removed.

The proportion of individuals for whom an anaphylaxis event was recorded in both Ambulance and EDDC was determined as the count of anaphylaxis events in each dataset by year and the proportion occurring in both datasets (represented as a percentage).

To investigate the significance of the change in anaphylaxis rates over time, a standard Poisson regression model was fitted to the counts of anaphylaxis events, with year modelled as a continuous covariate, and the log of rescaled population denominator (so that rates are estimated per 10^5^ population) modelled as an offset term. The average percent change was calculated from the beta coefficient for year from the fitted Poisson regression model. All statistical analyses were performed using R (version 3.5.1 for Windows).^15^

## Results

We identified 16,269 individuals in the WAAC, representing 187,351 anaphylaxis and other health events in any of HMDC, EDDC, Ambulance or Deaths datasets for 1980–2014. Of these, we examined a subset of 12,637 individuals (15,462 anaphylaxis events), for the period 2002–2013 (Fig. 1). The mean (SD) anaphylaxis age was relatively stable across the time period at 31.8 (21.6) years, with the minimum age being zero years, and the maximum being 100 years. On average, males and females were evenly distributed across the time period (females, 7642/15,462; 49.6%). Most anaphylaxis events were identified in the emergency (EDDC) dataset (11,290/15,462; 73.0% of events) and/or from hospital admissions (HMDC; 7559/15,462; 48.9% of events). In one-fifth of events, patients were attended by ambulance (3045/15,462; 19.7%). A total 25 deaths were recorded due to anaphylaxis (25/15,462; 0.2% of events). The majority of anaphylaxis events were coded as unspecified anaphylaxis (11,756/15,462; 76.0%), and represented people living in areas of greater socioeconomic advantage (quintile 5, 8020/13,475 with recorded SEIFA; 59.5%). Almost all events were in non-Indigenous patients (14,958/15,462; 97.5%), (Table 1; and detailed data by year in “Table X1” in the Supplemental Material).

**Table 1:** Characteristics of the 2002–2013 subset of the Western Australian Anaphylaxis Cohort

Category		Year			
		2002	2007	2013	Total 2002–2013
Anaphylaxis events^a^	n	303	1155	2060	15,462
Age	(years) mean (SD)	33.9 (23.4)	32.9 (22.0)	32.3 (22.0)	31.8 (21.6)
Min age	(years)	0	0	0	0
Max age	(years)	90	99	100	100
Gender					
Female	n (%)	139 (46.0)	555 (48.1)	1043 (50.9)	7642 (49.6)
Cause^b^					
Food	n (%)	41 (13.5)	57 (4.9)	164 (8.0)	1020 (6.6)
Medication	n (%)	52 (17.2)	187 (16.2)	315 (15.3)	2595 (16.8)
Unspecified	n (%)	202 (66.7)	904 (78.3)	1575 (76.5)	11,756 (76.0)
Indigenous Status					
Not Indigenous	n (%)	292 (96.7)	1126 (98.6)	1988 (97.4)	14,958 (97.5)
SEIFA IRSAD^c^					
Quintile 1	n (%)	4 (1.6)	4 (0.4)	12 (0.7)	99 (0.7)
Quintile 2	n (%)	5 (2.0)	9 (0.9)	26 (1.5)	205 (1.5)
Quintile 3	n (%)	29 (11.5)	57 (5.5)	163 (9.5)	1073 (8.0)
Quintile 4	n (%)	65 (25.8)	311 (30.1)	471 (27.4)	4078 (30.3)
Quintile 5	n (%)	149 (59.1)	651 (63.1)	1046 (60.9)	8020 (59.5)
Dataset^d^					
Ambulance	n	65	165	539	3045
EDDC	n	93	938	1396	11,290
HMDC	n	218	521	1076	7559
Deaths	n	1	6	1	25

SEIFA: Socio-Economic Indexes for Areas.

IRSAD: Index of Relative Socio-Economic Advantage and Disadvantage.

HMDC: Hospital Morbidity Data Collection.

EDDC: Emergency Department Data Collection.

a From merged datasets (Ambulance, EDDC, HMDC, Deaths). Count accounts for transfer exclusions and thus represents separate anaphylaxis events in the combined dataset.

b Cause unspecified includes ICD-coded 78.2 or equivalent and all ambulance events (where cause could not be distinguished).

c Denominator is 13,475. There were 1987 records missing SEIFA values.

d Count represents anaphylaxis event interaction with the dataset. Cases may be present in more than one dataset for the same event

### Annual anaphylaxis event rates

**Fig. 2:**
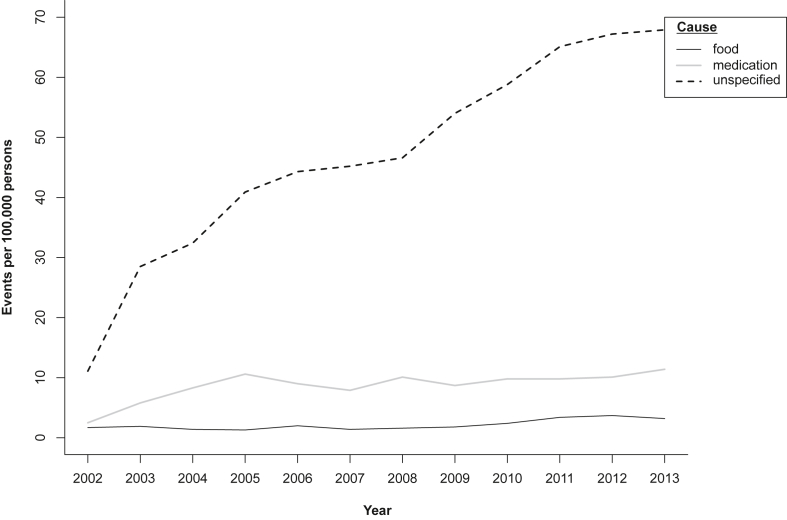
**Annual rates of anaphylaxis events in Western Australia, by different causes,** Food: solid black line, Medication: grey line, Unspecified: dashed line

Total (all cause, all ages and genders) anaphylaxis rates increased from 15.4 to 82.5/10^5^ population between 2002 and 2013. Over the same period, food-related anaphylaxis increased from 1.7 to 3.2/10^5^, medication-related anaphylaxis from 2.5 to 11.4/10^5^, and unspecified anaphylaxis from 11.1 to 67.9/10^5^ (Table 2, Fig. 2; and detailed data by year in “Table X2” in the Supplemental Material).

**Table 2:** Anaphylaxis event rates, with percentage and fold change, 2002–2013

Category	Merged datasets (Ambulance, EDDC, HMDC, Deaths)		Number of anaphylaxis events per 100,000 persons by year^a^			Change				
			2002	2007	2013	% change 2002–2013	fold-change 2002–2013	Average annual % increase across all years 2002–2013	95% CI	p-value for the change
**All persons**	**All ages**	Food	1.7	1.4	3.3	90.1	1.9	9.2	(6.6–12.0)	<0.001
		Medication	2.5	7.9	11.4	346.9	4.5	5.8	(4.5–7.1)	<0.001
		Unspecified	11.2	45.3	67.9	508.7	6.1	10.4	(9.8–11.0)	<0.001
**Males**	**Age (years)**	**All age, all cause**	**16.9**	**56.5**	**79.8**	**372.9**	**4.7**	**9.4**	**(8.7**–**10.2)**	**<0.001**
	0–4	Food	12.4	5.7	8.1	−34.3	0.7	2.5	(-3.5-9.0)	0.421
	0–4	Medication	1.6	7.2	8.1	424.5	5.2	13.1	(2.7–25.4)	0.015
	0–4	Unspecified	18.6	93.2	171.9	825.9	9.3	13.0	(10.9–15.2)	<0.001
	5–14	Food	2.8	2.1	4.4	55.8	1.6	15.7	(7.7–24.8)	<0.001
	5–14	Medication	0.7	1.4	4.4	521.1	6.2	11.3	(2.5–21.3)	0.012
	5–14	Unspecified	22.6	65.7	98.3	334.6	4.3	10.6	(8.8–12.5)	<0.001
	15–19	Food	1.4	3.9	2.5	78.3	1.8	4.7	(-7.0-18.4)	0.449
	15–19	Medication	2.8	9.1	8.6	211.6	3.1	7.1	(-0.4-15.5)	0.067
	15–19	Unspecified	6.9	35.3	83.5	1110.1	12.1	11.4	(8.4–14.5)	<0.001
	20–24	Food	1.5	2.5	2.1	44.5	1.4	0.1	(-11.6–13.7)	0.982
	20–24	Medication	1.5	5.0	8.4	477.4	5.8	4.6	(-2.6-12.5)	0.218
	20–24	Unspecified	14.6	57.6	71.6	389.2	4.9	8.0	(5.4–10.8)	<0.001
	25–34	Food	0.7	1.4	1.0	36.6	1.4	3.0	(-6.5-13.7)	0.553
	25–34	Medication	0.7	7.4	2.9	308.5	4.1	3.6	(-2.0-9.6)	0.214
	25–34	Unspecified	9.9	48.7	50.3	408.8	5.1	7.3	(5.2–9.5)	<0.001
	35–64	Food	0.3	0.9	1.7	534.6	6.3	9.5	(1.3–18.7)	0.025
	35–64	Medication	5.0	6.1	7.6	52.5	1.5	3.3	(0.4–6.3)	0.025
	35–64	Unspecified	9.5	38.8	58.2	516.3	6.2	10.7	(9.2–12.2)	<0.001
	≥65	Food	0.0	0.0	3.4	NA	NA	24.8	(6.1–51.4)	0.013
	≥65	Medication	2.1	15.7	15.8	665.5	7.7	9.8	(5.0–15.1)	<0.001
	≥65	Unspecified	9.3	31.4	32.9	254.5	3.5	7.8	(4.4–11.3)	<0.001
**Females**	**Age (years)**	**All age, all cause**	**14.5**	**53.1**	**84.6**	**485.3**	**5.9**	**9.6**	**(8.9**–**10.3)**	**<0.001**
	0–4	Food	9.7	3.0	11.0	12.9	1.1	7.2	(-1.2-16.8)	0.101
	0–4	Medication	0.0	3.0	2.4	NA	NA	−1.2	(-10.8–9.4)	0.812
	0–4	Unspecified	3.3	54.4	84.3	2493.8	25.9	12.4	(9.6–15.4)	<0.001
	5–14	Food	0.8	0.7	4.6	512.0	6.1	25.4	(10.5–44.7)	<0.001
	5–14	Medication	0.0	2.2	2.6	NA	NA	2.3	(-6.5-12.2)	0.619
	5–14	Unspecified	10.5	44.9	62.3	495.4	6.0	12.8	(10.3–15.4)	<0.001
	15–19	Food	2.9	1.4	6.6	126.1	2.3	15.8	(3.8–30.4)	0.011
	15–19	Medication	1.5	5.5	7.9	444.8	5.4	7.8	(0.2–16.3)	0.047
	15–19	Unspecified	13.1	52.6	104.0	695.9	8.0	11.9	(9.1–14.8)	<0.001
	20–24	Food	0.0	1.4	3.4	NA	NA	7.2	(-3.9-20.3)	0.219
	20–24	Medication	3.1	10.8	15.8	409.7	5.1	0.6	(-4.5-6.1)	0.813
	20–24	Unspecified	7.7	63.4	85.5	1007.6	11.1	10.7	(8.0–13.6)	<0.001
	25–34	Food	1.4	1.4	4.2	191.0	2.9	18.1	(6.3–32.6)	0.003
	25–34	Medication	2.2	7.0	16.8	680.5	7.8	8.8	(4.5–13.4)	<0.001
	25–34	Unspecified	6.5	52.0	79.2	1125.4	12.3	9.6	(7.6–11.6)	<0.001
	35–64	Food	1.1	0.7	2.3	115.0	2.1	8.5	(1.6–16.0)	0.017
	35–64	Medication	3.5	11.5	21.8	527.4	6.3	6.2	(3.9–8.6)	<0.001
	35–64	Unspecified	11.7	37.5	56.5	381.8	4.8	9.5	(8.1–11.0)	<0.001
	≥65	Food	1.7	1.5	3.0	79.8	1.8	7.2	(-5.4-22.4)	0.288
	≥65	Medication	2.5	13.4	15.7	520.6	6.2	4.7	(1.0–8.7)	0.014
	≥65	Unspecified	10.9	24.5	39.3	258.8	3.6	9.8	(6.6–13.1)	<0.001

HMDC: Hospital Morbidity Data Collection.

EDDC: Emergency Department Data Collection.

a Event rates exclude transfers and thus represent separate anaphylaxis events. Individuals may have more than one event within and/or across time periods.

### Impact of age, gender, cause, and dataset on anaphylaxis event rates

Anaphylaxis event rates increased significantly in males from 16.9 to 79.8/10^5^, and in females from 14.4 to 84.6/10^5^ population, between 2002 and 2013, both p < 0.001; Table 2. Unspecified anaphylaxis accounted for the greatest increase in both genders, with the highest rates observed in 2013, in males 0–4 years (171.9/10^5^), and in females 15–19 years (104.0/10^5^). For food-related anaphylaxis, both males and females 0–4 years showed the highest rates (8.1 and 11.0/10^5^ respectively), while medication-related anaphylaxis was highest in males≥65 years (15.8/10^5^) and females 35–64 years (21.8/10^5^), all in 2013 (Table 2).

**Fig. 3:**
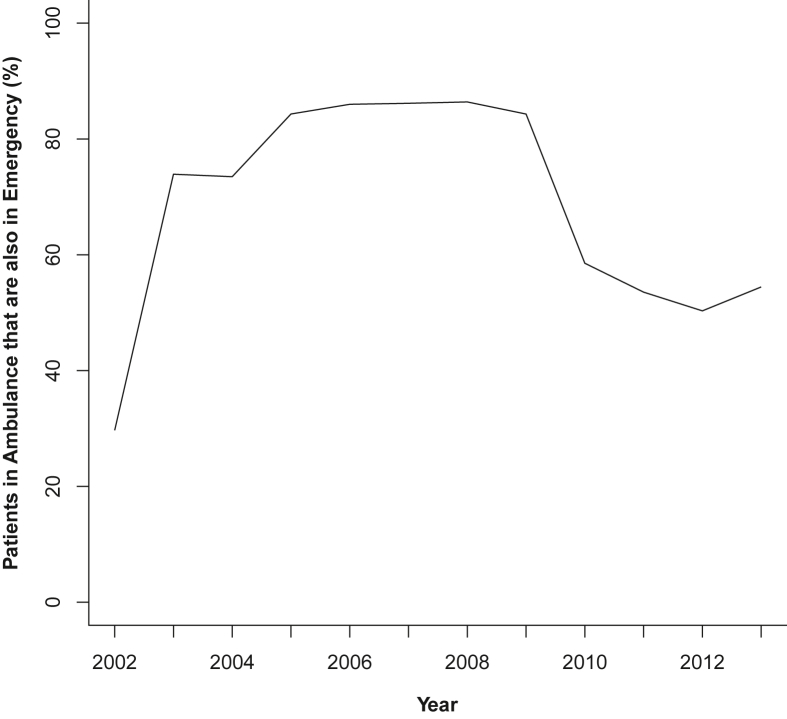
Patients with an event recorded in both the Ambulance and Emergency Department linked datasets, by year

**Fig. 4:**
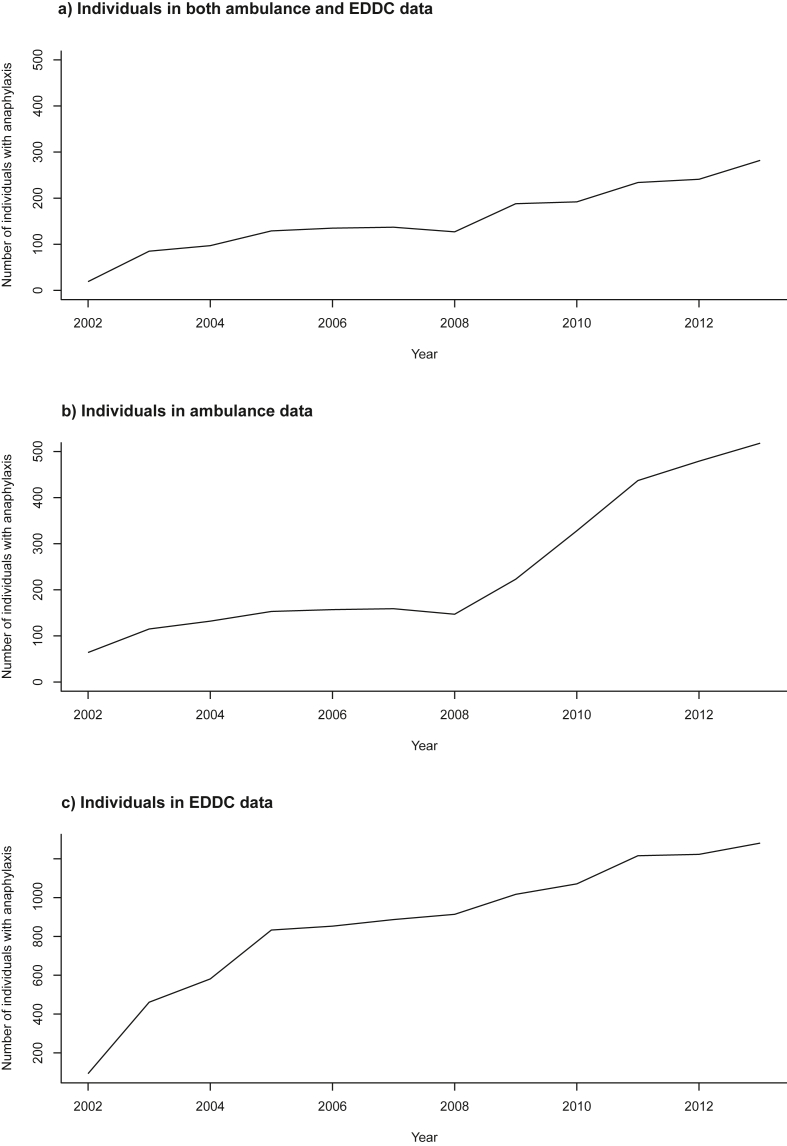
**Numbers of individuals with anaphylaxis in Western Australia, by dataset,** EDDC: Emergency Department Data Collection

A significant increase in anaphylaxis rates 2002–2013 (all cause, all ages and genders), was observed in Ambulance, EDDC and HMDC datasets (all p < 0.001), but not in the Deaths dataset; Table 3. The highest rates as seen in 2013, in the EDDC were 56.0/10^5^ population, followed by HMDC (43.2/10^5^) and Ambulance datasets (21.6/10^5^). The proportion of individuals for whom an anaphylaxis event was recorded in both Ambulance and EDDC datasets was high between 2002 and 2009 (73.9–84.3%), but decreased in 2010 (58.5%), and remained stable to 2013 (54.4%) (Table 4 and Fig. 3, Fig. 4). Only 25 anaphylaxis-related deaths were recorded in Deaths for the entire WAAC (1980–2014; all during 2002–2013). Between 2002 and 2013, 2 deaths were attributed to food, 1 to medication, and 22 were coded as unspecified anaphylaxis (Table 1, Table 3; detailed data by year in “Table X1” and “Table X3” in the Supplemental Material).

**Table 3:** Anaphylaxis event rates, with percentage and fold change 2002–2013, by dataset

Category		Number of anaphylaxis events per 100,000 persons by year^a^			Change				
		2002	2007	2013	% change 2002–2013	fold-change 2002–2013	Average annual % increase across all years 2002–2013	95% CI	p-value for the change
**Dataset**	**Cause**								
HMDC	All	11.3	24.74	43.16	281.9	3.8	11.8	(11.0–12.6)	<0.001
	Food	2.64	7.93	16.13	511	6.1	16.3	(14.9–17.8)	<0.001
	Medication	2.96	5.32	10.55	256.4	3.6	10.2	(8.7–11.8)	<0.001
	Unspecified	5.44	11.21	16.21	198	3	9.5	(8.3–10.7)	<0.001
EDDC	All	4.82	44.54	56	1061.8	11.6	9.0	(8.4–9.6)	<0.001
	Food	0	0	0.52	Not defined	Not defined	167.4	(70.0–423.3)	<0.001
	Medication	0.05	5.7	5.17	10240	103.4	3.0	(1.5–4.6)	<0.001
	Unspecified	4.61	38.7	50.22	989.4	10.9	9.9	(9.3–10.6)	<0.001
Ambulance	Unspecified	3.37	7.83	21.62	541.5	6.4	16.4	(15.1–17.7)	<0.001
Death	All	0.05	0.28	0.04	−20	0.8	3.2	(-7.9-16.0)	0.594
	Food	0	0	0	Not defined	1	61.0	(-6.0-387.7)	0.206
	Medication	0	0	0	Not defined	1	43.4	(-25.4–502.2)	0.411
	Unspecified	0.05	0.28	0.04	−20	0.8	−0.5	(-11.9–12.4)	0.931

HMDC: Hospital Morbidity Data Collection.

EDDC: Emergency Department Data Collection.

a Event rates are dataset specific and thus the same anaphylaxis event may be represented across more than one dataset in a given period. Individuals may have more than one event within and/or across time periods

**Table 4:** Proportion of individuals recorded in Ambulance and EDDC datasets for the same anaphylaxis event.

Year	Ambulance and EDDC (n)	Ambulance only (n)	Proportion in both datasets (%)
2002	19	64	29.7
2003	85	115	73.9
2004	97	132	73.5
2005	129	153	84.3
2006	135	157	86.0
2007	137	159	86.2
2008	127	147	86.4
2009	188	223	84.3
2010	192	328	58.5
2011	234	437	53.6
2012	241	479	50.3
2013	282	518	54.4

EDDC: Emergency Department Data Collection

### Change in anaphylaxis event rates

Total anaphylaxis events (all ages and genders) to food increased 1.9-fold, with a 4.5-fold increase in medication-related anaphylaxis and a 6.1-fold increase attributed to unspecified causes; all 2002–2013 (Table 2). The average annual percent increase (95% CI) for food-related anaphylaxis was 9.2% (6.6–12.0); for medication-related anaphylaxis was 5.8% (4.5–7.1); and for unspecified anaphylaxis was 10.4% (9.8–11.0); all p < 0.001.

Table 2 shows that for males alone, all age, all cause anaphylaxis increased 4.7-fold during 2002–2013 (average annual increase 9.4% [8.7–10.2]), with a 5.9-fold increase for females over the same time (average annual increase 9.6% [8.9–10.3]). The greatest change in anaphylaxis event rates 2002–2013 was seen in females 0–4 years, for unspecified anaphylaxis (25.9 fold increase, average annual increase 12.4% [9.6–15.4]), followed by females 25–34, also for unspecified anaphylaxis (12.3 fold increase, average annual increase 9.6% [7.6–11.6]), and males 15–19 years, again for unspecified anaphylaxis (12.1 fold increase, average annual increase 11.4% [8.4–14.5]).

## Discussion

The Epidemiology of Anaphylaxis in Western Australia (EPAWA) study is the first study worldwide to combine population-level ambulance, emergency department, hospital inpatient admissions, and mortality data to identify an anaphylaxis cohort to facilitate a comprehensive description of the epidemiology of anaphylaxis at the population level.

The 2002–2013 WAAC subset reported in this phase of EPAWA represents 12,637 individuals who experienced 15,462 anaphylaxis events across the 4 datasets. Anaphylaxis occurred across all ages (0–100 years, mean 31.8 years), predominately in non-Indigenous and in people living in areas of greater socioeconomic advantage, with a high proportion of events (73%) treated in an emergency department. Almost half of the anaphylaxis patients in the WAAC were identified in the inpatient admissions (HMDC) dataset. This proportion is different to other population-based studies, which show 5–20% of anaphylaxis patients are admitted.^2^^,^^4^^,^^16^^,^^17^ The HMDC dataset includes patients admitted to metropolitan hospitals as inpatients; to emergency department observation units; and to hospitals where there is no emergency department, such as those outside the major metropolitan areas in Western Australia. The observed difference may reflect the unique remoteness of many parts of Western Australia, where there is a lack of access to urgent care, and thus a preference for observation rather than discharge after treatment of an anaphylaxis event. It may also reflect anaphylaxis events arising during admission for other causes, or a propensity in Western Australia to admit a high proportion of cases. Moreover, this raises questions as to whether we have indeed captured all anaphylaxis events in patients who are not admitted, as a greater number of events not admitted to hospital would result in a lower proportion of admissions.

Anaphylaxis events increased markedly over the study period: females had more anaphylaxis events and a higher increase in rates overall than males, although the broad distribution of events varied by age and gender. Indeed, males experienced more anaphylaxis across the youngest age groups, whereas females experienced more anaphylaxis in teen and adult age groups. While the gender distribution is similar to past research,^4^^,^^5^^,^^18^ our rates and change in rates are considerably higher across all ages and genders than previously reported anaphylaxis estimates both in Australia (events of 17.7/10^5^ population, 2011–2012^6^) and beyond (incidence of 7–42/10^5^ person-years^5^^,^^9^^,^^17^, ^18^, ^19^).

Given past estimates are based on admissions data, emergency department data and mostly single population datasets, we expect our rates to be higher as more cases are likely to be identified from the different sources. Yet, considering our admissions data alone, Western Australian anaphylaxis hospital admission rates of 43.2/10^5^ population are more than double previously reported Australian anaphylaxis hospital admission rates, for the same time period.^6^ This raises questions about coding, identification and reporting of anaphylaxis, and whether there is a genuinely higher rate of anaphylaxis events in Western Australia compared to the rest of the nation. There may be differences in health seeking behaviour in Western Australia that are unique to this state or a greater propensity by clinical coders to code anaphylaxis based on symptoms. Regardless, our results are more consistent with other linked data studies, where incidence rates of 21–49.8/10^5^ person-years were reported.^20^, ^21^, ^22^ These rates represent only new cases, and although our event rates represent all anaphylaxis encounters (and include recurrent events), our 12,637 individuals experiencing 15,462 events suggests that for the vast majority, their anaphylaxis is a one-off event.

### Impact of anaphylaxis cause

In addition to all ambulance events, unspecified anaphylaxis represented almost 100% of events in emergency and deaths data, and 50% of all events in admissions data, far greater than that observed in past population-based research of emergency (40–57%),^5^^,^^18^ inpatient admissions (33–38%)^6^^,^^18^ and general practice (37%)^23^ datasets. This highlights a key issue in coding and may represent issues with identification of anaphylaxis cause at the time of interaction with the hospital system in Western Australia.

Medication-related anaphylaxis increased 4.5-fold in our study, with total and hospital admission rates substantially higher than past Australian admissions data estimates (11.4 and 10.5 versus 4.3/10^5^ population respectively).^6^ It is expected that admissions rates (and indeed previous estimates) include events arising during non-anaphylaxis admissions (where medications are administered as part of that admission, eg, anaesthetic agents, intravenous antibiotics). However, the reasons for our observed 103-fold increase in emergency department presentations and much higher rates for admissions for medication-related anaphylaxis are uncertain. They may represent a true increase in medication allergy, increased community exposure in previously sensitised patients through greater access to over-the-counter medications such as non-steroidal anti-inflammatory agents, or altered recognition and coding. Despite these differences, the highest rates of anaphylaxis in this study were seen in older adults, consistent with other research.^6^

Food-related anaphylaxis event rates were in general substantially lower in our study (3.2/10^5^ population in 2013) compared to other studies, including those where only 1 dataset was examined (range 8–9/10^5^ population),^5^^,^^6^ and where only paediatric cases were considered (range 2.4–140/10^5^ population).^24^, ^25^, ^26^, ^27^ Although food-related anaphylaxis event rates in the youngest population (0–4 years) appear to have stabilised (with low or negative percentage change from 2002 to 2013), this is likely an artefact of extremely high rates of unspecified anaphylaxis in this age group (up to 171.9/10^5^ population), especially given food allergy in Australia affects more than 10% of infants and 4% of children,^28^^,^^29^ and considering our annual percent growth of 11–13% in medication-related anaphylaxis in younger males.

The use of population level data in anaphylaxis epidemiology research has well recognised limitations in eliciting cause types.^30^^,^^31^ For example, our proportion of unspecified anaphylaxis was noticeably higher than that identified by alternate approaches, including case reviews from established datasets (such as emergency department records in a single hospital), where food is repeatedly identified as the most common causative agent (65–96% of cases).^26^^,^^27^^,^^32^ Such an approach allows a more granular examination of causes and provides richer detail than population level data can provide, but may be less generalisable and at the expense of breadth of population level epidemiology estimates. A compromise between these population dataset and case review approaches, such as that using purpose-built, registry-collected data^24^^,^^33^, ^34^, ^35^ offers greater detail at a population level, but these systems are expensive, resource-intensive and require political will to be implemented and sustained.

Mandatory reporting of anaphylaxis patients presenting to hospital for treatment commenced in the Australian state of Victoria in 2018, primarily as a notification system for public health action in the event of undeclared food allergens.^35^ Between November 2018 and May 2019 (26 weeks), 1200 notifications were received in this system, of which “unknown cause” of anaphylaxis accounted for 11%.^36^ By comparison, in 2013 (52 weeks), 1396 anaphylaxis events were identified in Western Australian emergency departments (in our study), and almost 100% of these were coded as unspecified anaphylaxis. Innately, the very process of making a mandatory report facilitates deeper consideration of the anaphylaxis cause, but this does not mean we should discount cases coded as unspecified: anaphylaxis is a clinical diagnosis based on circumstances of the reaction and reported symptoms, regardless of coding. It is difficult to find balance between the risks of misclassification (and missed classification) under the ICD coding system and case definition constraints in registries.

### Impact of dataset source

Beyond the contribution to more complete anaphylaxis event rates, the different datasets offer insight into the health seeking behaviour of patients experiencing anaphylaxis events. The highest interactions were seen in emergency and admissions datasets, with a remarkable 11.6-fold increase in emergency presentations (to 56.0/10^5^ population in 2013), substantially higher than previous estimates over a similar time period (ranging from 17.2 to 32/10^5^ population with fold increases less than 2^4^^,^^25^^,^^37^). Patients are encouraged to attend an emergency room for every anaphylaxis event, including after epinephrine administration and ambulance attendance, in case of protracted or biphasic anaphylaxis.^38^^,^^39^ Improved education and use of Anaphylaxis Action Plans may have contributed to this finding.^40^ Furthermore, ambulance attendance increased more than 6-fold (to 21.6/10^5^ population) over the study period, however, up to 45% of individuals experiencing anaphylaxis were not recorded with an anaphylaxis code in both ambulance and emergency datasets. This suggests patients may not have been transported to the emergency room after ambulance attendance, or if they were transported, they were subsequently coded with a non-anaphylaxis code in EDDC. Aside from data recording and/or coding issues, it is difficult without case-review to tease out whether this is a real effect, and if so, the potential reasons for this divergence from recommendations. Considering anaphylaxis cause was not identified in the ambulance dataset, up to half of the patients in our study may have had no further interaction with the health system, and may be unaware of their eliciting cause with no follow up to inform them. Closer investigation of ambulance transfers to hospital or otherwise is needed.

Despite high and increasing anaphylaxis presentations to emergency, admissions and ambulance datasets, only 25 anaphylaxis-coded deaths were identified during our study. Coding of the anaphylaxis cause was poor, with only 3 deaths specifically coded: food (2) or medication (1). Relying on ICD-10 coding has been shown to underestimate anaphylaxis deaths.^41^ While our rates are similar to those reported for similar time periods in the United States (0.063–0.076/10^5^ population^42^), United Kingdom (0.047/10^5^ population^9^) and Australia (0.099/10^5^ population^43^), the variability in our data and lack of specified cause highlights the need for the changes in coding that ICD-11 will bring.^44^

### Strengths and limitations

The EPAWA study linked administrative datasets for ambulance, emergency, hospital admissions and death registrations in order to provide the most comprehensive epidemiological capture of anaphylaxis cases to date. However, the absence of a dedicated general practice or other community database for linkage means our results do not include events managed solely in the primary care or community setting, or those for whom patients do not seek care, and may still underestimate the epidemiology of anaphylaxis in Western Australia.

A key advantage of linked health data is the ability to identify events in the same individual across different datasets and timeframes, to determine event rates, incidence, and recurrence.^45^ Anaphylaxis is an acute manifestation of a long-term condition with reactions interspaced by long event-free periods. Event rates are therefore important at the health system level to plan and provide services for the number of cases that are expected, regardless of whether they are repeat cases or not. Repeat cases are important from a systems perspective as they add to the total burden of anaphylaxis on the health system. This phase of the EPAWA study reports event rates without duplication across datasets and therefore provides a perspective of the overall burden of anaphylaxis in the Western Australian health system. However, incidence rates are important to distinguish the number of new cases from recurrent cases, and provide insight into how we should manage patients at the individual level. The next phase of the EPAWA study will determine incidence and recurrence rates for individuals newly diagnosed with anaphylaxis, based on a 25-year look back period.

The reliance on ICD codes to identify anaphylaxis events is a well-known limitation,^30^^,^^31^ although a necessary feature of research with large datasets. Compared to past research, higher rates of unspecified anaphylaxis were seen across all datasets in our study. We did not verify coding in any datasets, therefore it is not clear if the rates observed could have been coded with an alternate (anaphylaxis or non-anaphylaxis) code. Furthermore, it was not possible to distinguish a cause in the Ambulance dataset as this does not utilise the ICD system. The inclusion of ambulance cases (where a defined cause could not be identified from other linked datasets) along with ICD-coded unspecified anaphylaxis will have increased the overall rates of unspecified anaphylaxis. However this highlights the issues paramedics and doctors face around time, skills and testing capacity to necessarily identify a cause and reinforces the need for patients to be referred automatically to allergy specialist services after each event for evaluation and advice.

Finally, these findings relate to the period 2002–2013 and although they shed new light, it is recognised that anaphylaxis epidemiology may have changed since that time. The population datasets included in this study extend beyond 2013. However at the time of this research the common time period for which all datasets were linked was 2002–2013, so analyses were limited to this time period.

## Conclusions

Through the first phase of the EPAWA study we identified significantly higher rates of total anaphylaxis than previously reported, with extreme increases in anaphylaxis events between 2002 and 2013. While the combination of 4 population-level datasets provides a more comprehensive capture of cases, even at the individual dataset level, rates of anaphylaxis in Western Australia are substantially higher than those previously reported for similar time periods, both in Australia and worldwide. Population-based anaphylaxis research holds much promise. However to provide useful insight to healthcare planning, it is crucial that we better identify the drivers of anaphylaxis in administrative datasets, clarify the discrepancies between Western Australian and Australian anaphylaxis event rates, and consider avenues to incorporate anaphylaxis cases from the community into present epidemiological estimates.

## Financial support

The research was supported by a research grant provided by the Western Australian Department of Health (G05767) and an unrestricted grant provided by Mylan Specialty Limited (IIT16-007).

## Availability of data and materials

The Western Australian Department of Health approvals for data forbid release of linked data publicly, in line with privacy laws. Therefore data for this project is not available for release.

## Authors’ contributions

SMS conceptualized, designed and supervised the study, raised funding for the study, assisted with data analysis, interpreted the results, wrote the first draft and prepared the final version of the manuscript. RossJM assisted in data collection, performed data analysis, interpreted the results, and reviewed the manuscript. KM assisted with data collection and data analysis, interpreted the results, and reviewed the manuscript. SLS assisted with background review, interpreted the results and reviewed the manuscript. PB assisted with access to data and data collection, interpreted the results and reviewed the manuscript. RaymondJM provided intellectual input, interpreted the results and reviewed the manuscript. FMS conceptualized, designed and supervised the study, assisted with data analysis, interpreted the results and reviewed the manuscript. All authors contributed to the interpretation and discussion of the results and have read and approved the final version of the manuscript.

## Ethics statement

Approval to conduct this research was obtained from the Human Research Ethics Committees of the Western Australian Department of Health and The University of Western Australia.

## Submission declaration

The work described has not been published previously, is not under consideration for publication elsewhere, its publication is approved by all authors and tacitly or explicitly by the responsible authorities where the work was carried out, and if accepted, it will not be published elsewhere in the same form, in English or in any other language, including electronically without the written consent of the copyright holder.

## Disclaimer

The views expressed in the submitted article represent the original interpretations and opinions of the authors and are not an official position of any affiliated institution or funder.

## Declaration of competing interest

Potential Competing Interests: The authors report no competing interests.

## References

[1] Simons F.E.R., Ardusso L.R., Bilò M.B. (2011). World allergy organization guidelines for the assessment and management of anaphylaxis. World Allergy Organ J.

[2] Bohlke K., Davis R.L., DeStefano F. (2004). Epidemiology of anaphylaxis among children and adolescents enrolled in a health maintenance organization. J Allergy Clin Immunol.

[3] Lin R.Y., Anderson A.S., Shah S.N., Nurruzzaman F. (2008). Increasing anaphylaxis hospitalizations in the first 2 decades of life: New York State, 1990 -2006. Ann Allergy Asthma Immunol.

[4] Motosue M.S., Bellolio M.F., Van Houten H.K., Shah N.D., Campbell R.L. (2018). National trends in emergency department visits and hospitalizations for food-induced anaphylaxis in US children. Pediatr Allergy Immunol.

[5] Motosue M.S., Bellolio M.F., Van Houten H.K., Shah N.D., Campbell R.L. (2016). Increasing emergency department visits for anaphylaxis, 2005-2014. J Allergy Clin Immunol Pract.

[6] Mullins R.J., Dear K.B., Tang M.L. (2015). Time trends in Australian hospital anaphylaxis admissions in 1998-1999 to 2011-2012. J Allergy Clin Immunol.

[7] Poulos L.M., Waters A.M., Correll P.K., Loblay R.H., Marks G.B. (2007). Trends in hospitalizations for anaphylaxis, angioedema, and urticaria in Australia, 1993-1994 to 2004-2005. J Allergy Clin Immunol.

[8] Sheikh A., Alves B. (2000). Hospital admissions for acute anaphylaxis: time trend study. BMJ.

[9] Turner P.J., Gowland M.H., Sharma V. (2015). Increase in anaphylaxis-related hospitalizations but no increase in fatalities: an analysis of United Kingdom national anaphylaxis data, 1992-2012. J Allergy Clin Immunol.

[10] Wang Y., Allen K.J., Suaini N.H.A., McWilliam V., Peters R.L., Koplin J.J. (2019). The global incidence and prevalence of anaphylaxis in children in the general population: a systematic review. Allergy.

[11] Kelman C.W., Bass A.J., Holman C.D.J. (2002). Research use of linked health data – a best practice protocol. Aust NZ J Public Health.

[12] Holman C.D.J., Bass A.J., Rosman D.L. (2008). A decade of data linkage in Western Australia: strategic design, applications and benefits of the WA data linkage system. Aust Health Rev.

[13] Australian Bureau of Statistics Socio-economic Indexes for areas (SEIFA). http://www.abs.gov.au/websitedbs/censushome.nsf/home/seifa.

[14] Australian Bureau of Statistics Estimated resident population by single year of age, Australia. Catalogue Number 31010. data cube: excel spreadsheet, cat. no. 3101.0. http://www.abs.gov.au/AUSSTATS/abs@.nsf/DetailsPage/3101.0Mar%202018?OpenDocument.

[15] The R Foundation for Statistical Computing R - a language and environment for statistical computing. https://www.R-project.org.

[16] Grabenhenrich L.B., Dolle S., Moneret-Vautrin A. (2016). Anaphylaxis in children and adolescents: the European anaphylaxis registry. J Allergy Clin Immunol.

[17] Lee S., Hess E.P., Lohse C., Gilani W., Chamberlain A.M., Campbell R.L. (2017). Trends, characteristics, and incidence of anaphylaxis in 2001-2010: a population-based study. J Allergy Clin Immunol.

[18] Harduar-Morano L., Simon M.R., Watkins S., Blackmore C. (2011). A population-based epidemiologic study of emergency department visits for anaphylaxis in Florida. J Allergy Clin Immunol.

[19] Kivisto J.E., Protudjer J.L., Karjalainen J., Wickman M., Bergstrom A., Mattila V.M. (2016). Hospitalizations due to allergic reactions in Finnish and Swedish children during 1999-2011. Allergy.

[20] Decker W.W., Campbell R.L., Manivannan V. (2008). The etiology and incidence of anaphylaxis in Rochester, Minnesota: a report from the Rochester Epidemiology Project. J Allergy Clin Immunol.

[21] Lee S., Bashore C., Lohse C.M. (2016). Rate of recurrent anaphylaxis and associated risk factors among Olmsted County, Minnesota, residents: a population-based study. Ann Allergy Asthma Immunol.

[22] Yocum M.W., Butterfield J.H., Klein J.S., Volcheck G.W., Schroeder D.R., Silverstein M.D. (1999). Epidemiology of anaphylaxis in Olmsted County: a population-based study. J Allergy Clin Immunol.

[23] Gonzalez-Perez A., Aponte Z., Vidaurre C.F., Rodriguez L.A. (2010). Anaphylaxis epidemiology in patients with and patients without asthma: a United Kingdom database review. J Allergy Clin Immunol.

[24] Cohen N., Capua T., Pivko-Levy D., Ben-Shoshan M., Rimon A., Benor S. (2019). Improved diagnosis and treatment of anaphylaxis in a pediatric emergency department (2013-2018). J Allergy Clin Immunol Pract.

[25] Dyer A.A., Lau C.H., Smith T.L., Smith B.M., Gupta R.S. (2015). Pediatric emergency department visits and hospitalizations due to food-induced anaphylaxis in Illinois. Ann Allergy Asthma Immunol.

[26] Nogic C., Belousoff J., Krieser D. (2016). The diagnosis and management of children presenting with anaphylaxis to a metropolitan emergency department: a 2-year retrospective case series. J Paediatr Child Health.

[27] Speakman S., Kool B., Sinclair J., Fitzharris P. (2018). Paediatric food-induced anaphylaxis hospital presentations in New Zealand. J Paediatr Child Health.

[28] Loke P., Koplin J., Beck C. (2016). Statewide prevalence of school children at risk of anaphylaxis and rate of adrenaline autoinjector activation in Victorian government schools, Australia. J Allergy Clin Immunol.

[29] Osborne N.J., Koplin J.J., Martin P.E. (2011). Prevalence of challenge-proven IgE-mediated food allergy using population-based sampling and predetermined challenge criteria in infants. J Allergy Clin Immunol.

[30] Tanno L.K., Bierrenbach A.L., Simons F.E.R. (2018). Critical view of anaphylaxis epidemiology: open questions and new perspectives. Allergy Asthma Clin Immunol.

[31] Turner P.J., Campbell D.E. (2016). Epidemiology of severe anaphylaxis: can we use population-based data to understand anaphylaxis?. Curr Opin Allergy Clin Immunol.

[32] Wright C.D., Longjohn M., Lieberman P.L., Lieberman J.A. (2017). An analysis of anaphylaxis cases at a single pediatric emergency department during a 1-year period. Ann Allergy Asthma Immunol.

[33] Acker W.W., Plasek J.M., Blumenthal K.G. (2017). Prevalence of food allergies and intolerances documented in electronic health records. J Allergy Clin Immunol.

[34] Le M., Gabrielli S., Clarke A. (2019). Emergency management of anaphylaxis due to an unknown trigger: an 8-year follow-up study in Canada. J Allergy Clin Immunol Pract.

[35] Zubrinich C., Douglass J., Bartlett J., Patel M., Hew M. (2019). Anaphylaxis presentations to the emergency department: impending Victorian reporting legislation. Intern Med J.

[36] Clifford E. (2019). Department of Health and Human Services Victoria: Victoria's Anaphylaxis Notification System. http://allergenbureau.net/wp-content/uploads/2019/05/CLIFFORD_2019-Presentation-Anaphylaxis-Notification-System-FAMS2019.pdf.

[37] Vetander M., Helander D., Flodstrom C. (2012). Anaphylaxis and reactions to foods in children-a population-based case study of emergency department visits. Clin Exp Allergy.

[38] Alvarez-Perea A., Tanno L.K., Baeza M.L. (2017). How to manage anaphylaxis in primary care. Clin Transl Allergy.

[39] Fischer D., Vander Leek T.K., Ellis A.K., Kim H. (2018). Anaphylaxis. Allergy Asthma Clin Immunol..

[40] Simons F.E. (2009). Anaphylaxis: recent advances in assessment and treatment. J Allergy Clin Immunol.

[41] Tanno L., Ganem F., Demoly P., Toscano C., Bierrenbach A. (2012). Undernotification of anaphylaxis deaths in Brazil due to difficult coding under the ICD-10. Allergy.

[42] Ma L., Danoff T.M., Borish L. (2014). Case fatality and population mortality associated with anaphylaxis in the United States. J Allergy Clin Immunol.

[43] Mullins R.J., Wainstein B.K., Barnes E.H., Liew W.K., Campbell D.E. (2016). Increases in anaphylaxis fatalities in Australia from 1997 to 2013. Clin Exp Allergy.

[44] Tanno L.K., Chalmers R., Bierrenbach A.L. (2019). Changing the history of anaphylaxis mortality statistics through the world health organization's international classification of diseases-11. J Allergy Clin Immunol.

[45] Boyd J.H., Randall S.M., Ferrante A.M. (2015). Accuracy and completeness of patient pathways-the benefits of national data linkage in Australia. BMC Health Serv Res.

