# Supplementary material for: Increasing anaphylaxis events in Western Australia identified using four linked administrative datasets
Source: World Allergy Organ J. 2020 Nov 13;13(11):100480. doi: 10.1016/j.waojou.2020.100480 (PMC7677753; doi:10.1016/j.waojou.2020.100480)
Supplement: Multimedia component 1 [file mmc1.pdf]

Increasing anaphylaxis events in Western Australia identified using four linked administrative datasets

July 2020

Supplemental Appendix 1

<u>Supplemental Methods: Identification of transfer records and removal of duplicates for obtaining accurate counts of anaphylaxis events.</u>

A series of rules (Table E1) was developed to identify records pertaining to the same event (transfers): (i) records for the same patient were contiguous in time; and (ii) a code in one or both records verified the specific transfer (*verification codes*, Table E2). 'Contiguous in time' was defined for this study as records with the same date. However, emergency department presentation dates and dates of ambulance calls were available only at timescales of months or years, respectively.

Hence, transfer replicates were identified and excluded using a systematic approach, which accounts for limitations in the resolution of date information in the emergency department and ambulance datasets. In the first instance, exact dates in the inpatients or deaths datasets were used to identify transfer replicate rows. Then, replicate ambulance-emergency transfer records were consistently taken as the most recent (i.e., the latest month in the year) of those in the emergency dataset, for that patient in that year. Finally, any record that had been identified as a transfer replicate (i.e., any type of transfer) was excluded.

Table E1. Rules for identifying anaphylaxis transfer records in linked administrative data.*

	Transfer type (Dataset names[a])		Verification code[b]				Count 2002-2013
			Dataset 1		Dataset 2		
Rule	Dataset 1	Dataset 2	Variable	Code(s)	Variable	Code(s)	n
I	Ambulance	Emergency	-	-	arr_type	3	1,797
II	Ambulance	Inpatients	-	-	sor_tran	2 or 3	1,125
III	Emergency	Inpatients	disposal	1 or 3	sor_prof	4	2,022
IV	Inpatients		mos	1			71
V	Inpatients	Emergency	mos	1	visit type	15	19
VI	Emergency	Deaths	disposal	6 or 7	-	-	1
VII	Inpatients	Deaths	mos	8	-	-	7

Rules were developed for only those types of transfer where a verification code could be used, in addition to date (or month or year) information, for identifying the same patient events. Counts of transfer records are presented only for years where anaphylaxis data from all four datasets were available (calendar years 2002 to 2013).

[a] Dataset names correspond with the source (Dataset 1) and destination (Dataset 2) for episodes of care associated with each different type of transfer.

[b] 'arr_type' = Arrival Type; 'disposal' = Disposal Code; 'mos' = Mode of Separation; 'sor_prof' = Source of Referral – Professional; 'sor_tran' = Source of Referral – Transport. Code definitions provided in Table E2.

Table E2: Verification codes for identifying transfer records[c].

Rule[d]	Dataset	Variable	Code used	Value
I	Emergency	Arrival Type	3	Ambulance
II	Inpatients	Source of Referral - Transport	2	Ambulance - patient transport
II	Inpatients	Source of Referral – Transport	3	Ambulance - emergency
III	Emergency	Disposal Code	1	Admitted to ward/other admitted patient unit
III	Emergency	Disposal Code	3	Transferred to another hospital for admission
III	Inpatients	Source of Referral - Professional	4	Emergency Department Clinician
IV, V	Inpatients	Mode of Separation	1	Discharge/transfer to an (other) acute hospital
V	Emergency	Visit Type	15	Transfer from other hospital
VI	Emergency	Disposal Code	6	Died in ED
VI	Emergency	Disposal Code	7	Dead on arrival, not treated in ED
VII	Inpatients	Mode of Separation	8	Deceased

[c] Further information and data dictionaries available at https://www.datalinkage-wa.org.au/downloads/dataset-information (accessed 16 November 2018): "Emergency Department Data Collection", "Hospital Morbidity Data Collection", "Death Registrations" for emergency department, hospital inpatients, death registry datasets, respectively.

[d]As listed in Table E1
