# Supplementary material for: Increasing anaphylaxis events in Western Australia identified using four linked administrative datasets
Source: World Allergy Organ J. 2020 Nov 13;13(11):100480. doi: 10.1016/j.waojou.2020.100480 (PMC7677753; doi:10.1016/j.waojou.2020.100480)
Supplement: Multimedia component 2 [file mmc2.pdf]

Table 1. Characteristics of the 2002-2013 subset of the Western Australian Anaphylaxis Cohort by year

		Year												
**Category**		**2002**	**2003**	**2004**	**2005**	**2006**	**2007**	**2008**	**2009**	**2010**	**2011**	**2012**	**2013**	**Total 2002-2013**
Anaphylaxis events*	n	303	711	840	1,071	1,140	1,155	1,273	1,456	1,634	1,846	1,973	2,060	15,462
Age	(years) mean (SD)	33.9 (23.4)	32.7 (21.4)	33.2 (20.8)	31.9 (21.6)	31.6 (20.9)	32.9 (22.0)	32.3 (21.2)	30.8 (21.0)	31.5 (21.9)	30.4 (22.2)	31.3 (21.7)	32.3 (22.0)	31.8 (21.6)
Min age	(years)	0	0	0	0	0	0	0	0	0	0	0	0	0
Max age	(years)	90	91	91	87	90	99	94	91	96	94	92	100	100
Gender														
Female	n (%)	139 (46.0)	351 (49.4)	441 (52.5)	548 (51.2)	559 (49.0)	555 (48.1)	656 (51.5)	690 (47.4)	780 (47.9)	885 (48.2)	995 (50.7)	1,043 (50.9)	7,642 (49.6)
Cause**														
Food	n (%)	41 (13.5)	47 (6.6)	40 (4.8)	40 (3.7)	67 (5.9)	57 (4.9)	64 (5.0)	86 (5.9)	115 (7.0)	142 (7.7)	157 (8.0)	164 (8.0)	1,020 (6.6)
Medication	n (%)	52 (17.2)	132 (18.6)	190 (22.6)	253 (23.6)	205 (18.0)	187 (16.2)	237 (18.6)	229 (15.7)	250 (15.3)	262 (14.2)	283 (14.3)	315 (15.3)	2,595 (16.8)
Unspecified	n (%)	202 (66.7)	527 (74.1)	603 (71.8)	769 (71.8)	862 (75.6)	904 (78.3)	963 (75.6)	1,128 (77.5)	1,262 (77.2)	1,436 (77.8)	1,525 (77.3)	1,575 (76.5)	11,756 (76.0)
Indigenous Status														
Not Indigenous	n (%)	292 (96.7)	690 (97.5)	808 (97.8)	1,030 (97.9)	1,096 (97.7)	1,126 (98.6)	1,227 (96.5)	1,432 (98.5)	1,585 (97.5)	1,777 (96.9)	1,907 (97.3)	1,988 (97.4)	14,958 (97.5)
SEIFA Score														
SEIFA 1	n (%)	4 (1.6)	4 (0.6)	10 (1.3)	2 (0.2)	10 (1.0)	4 (0.4)	9 (0.8)	8 (0.6)	4 (0.3)	15 (1.0)	17 (1.0)	12 (0.7)	99 (0.7)
SEIFA 2	n (%)	5 (2.0)	26 (4.1)	11 (1.5)	13 (1.3)	13 (1.3)	9 (0.9)	12 (1.0)	18 (1.3)	18 (1.3)	28 (1.8)	26 (1.6)	26 (1.5)	205 (1.5)
SEIFA 3	n (%)	29 (11.5)	59 (9.3)	58 (7.8)	64 (6.6)	64 (6.4)	57 (5.5)	88 (7.5)	98 (7.3)	120 (8.5)	136 (8.7)	137 (8.4)	163 (9.5)	1,073 (8.0)
SEIFA 4	n (%)	65 (25.8)	197 (31.0)	260 (34.8)	295 (30.3)	345 (34.7)	311 (30.1)	333 (28.5)	394 (29.3)	425 (30.1)	455 (28.9)	527 (32.5)	471 (27.4)	4,078 (30.3)
SEIFA 5	n (%)	149 (59.1)	350 (55.0)	408 (54.6)	600 (61.6)	561 (56.5)	651 (63.1)	728 (62.2)	829 (61.5)	843 (59.8)	938 (59.7)	917 (56.5)	1,046 (60.9)	8,020 (59.5)
Dataset														
Ambulance***	n	65	120	138	163	166	165	153	234	345	451	506	539	3,045
EDDC***	n	93	489	620	871	897	938	1,024	1,146	1,187	1,294	1,335	1,396	11,290
HMDC***	n	218	297	331	411	530	521	563	716	875	987	1,034	1,076	7,559
Deaths***	n	1	2	1	0	2	6	2	1	2	4	3	1	25

* Merged datasets (Ambulance, EDDC, HMDC, Deaths) count accounts for transfer exclusions and thus represents separate anaphylaxis events in the combined dataset.
**Cause unspecified includes ICD-coded 78.2 or equivalent all Ambulance events (where cause is not distinguished in coding)
***Count represents anaphylaxis interaction with the dataset. Cases may be present in more than one dataset for the same event.
Records with missing variables were excluded from the relevant analysis: a total 40 events were missing age and gender; 126 events were missing Indigenous status; 1987 events were missing SEIFA score

SEIFA: Socio-Economic Indexes for Areas
HMDC: Hospital Morbidity Data Collection
EDDC: Emergency Department Data Collection
