# Supplementary material for: Increasing anaphylaxis events in Western Australia identified using four linked administrative datasets
Source: World Allergy Organ J. 2020 Nov 13;13(11):100480. doi: 10.1016/j.waojou.2020.100480 (PMC7677753; doi:10.1016/j.waojou.2020.100480)
Supplement: Multimedia component 3 [file mmc3.pdf]

Table 2. Anaphylaxis event rates, with percentage and fold change 2002-2013 by year

			Number of anaphylaxis events per 100,000 persons by year*												Change				
**Category**	**Merged datasets (Ambulance, EDDC, HMDC, Deaths)**		**2002**	**2003**	**2004**	**2005**	**2006**	**2007**	**2008**	**2009**	**2010**	**2011**	**2012**	**2013**	**% change 2002-2013**	**fold-change 2002-2013**	**Average annual % increase across all years 2002-2013**	**95% CI**	**p-value for the change**
**All persons**	**All ages**	Food	1.71	1.89	1.41	1.29	2.05	1.42	1.57	1.79	2.44	3.4	3.67	3.25	90.1	1.9	9.2	(6.6-12.0)	<0.001
		Medication	2.54	5.84	8.28	10.64	9.02	7.88	10.08	8.75	9.82	9.82	10.1	11.35	346.9	4.5	5.8	(4.5-7.1)	<0.001
		Unspecified	11.15	28.47	32.38	40.92	44.28	45.25	46.6	54.01	58.8	65.05	67.25	67.87	508.7	6.1	10.4	(9.8-11.0)	<0.001
**Males**	**Age (years)**	**All age, all cause**	**16.87**	**36.75**	**40.1**	**51.64**	**56.16**	**56.51**	**56.35**	**67.82**	**73.57**	**80.25**	**78.85**	**79.77**	**372.9**	**4.7**	**9.4**	**(8.7-10.2)**	**<0.001**
	0-4	Food	12.38	13.99	6.21	7.65	8.98	5.74	4.09	9.16	8.92	18.76	15.64	8.13	-34.3	0.7	2.5	(-3.5-9.0)	0.421
	0-4	Medication	1.55	0	0	7.65	1.5	7.17	4.09	5.24	6.37	2.5	6.02	8.13	424.5	5.2	13.1	(2.7-25.4)	0.015
	0-4	Unspecified	18.57	59.09	66.7	97.93	103.22	93.21	88.66	146.62	165.67	163.88	173.26	171.94	825.9	9.3	13.0	(10.9-15.2)	<0.001
	5-14	Food	2.83	1.41	1.41	1.4	2.78	2.08	3.44	4.07	8.08	5.96	9.05	4.41	55.8	1.6	15.7	(7.7-24.8)	<0.001
	5-14	Medication	0.71	0.71	2.11	4.21	2.78	1.38	2.75	2.03	4.71	3.97	3.88	4.41	521.1	6.2	11.3	(2.5-21.3)	0.012
	5-14	Unspecified	22.62	46.59	35.15	63.1	64.65	65.73	65.27	77.32	75.39	107.22	104.02	98.3	334.6	4.3	10.6	(8.8-12.5)	<0.001
	15-19	Food	1.38	5.47	1.36	0	0	3.92	3.85	1.27	2.53	2.53	4.97	2.46	78.3	1.8	4.7	(-7.0-18.4)	0.449
	15-19	Medication	2.76	2.74	4.07	10.71	3.98	9.14	6.41	8.86	5.07	10.11	7.45	8.6	211.6	3.1	7.1	(-0.4-15.5)	0.067
	15-19	Unspecified	6.9	28.72	43.43	44.2	59.73	35.26	28.2	64.52	67.12	83.37	59.6	83.5	1110.1	12.1	11.4	(8.4-14.5)	<0.001
	20-24	Food	1.46	4.29	0	2.7	2.6	2.51	0	3.4	2.22	2.18	2.13	2.11	44.5	1.4	0.1	(-11.6-13.7)	0.982
	20-24	Medication	1.46	1.43	4.17	9.44	9.11	5.01	14.37	10.21	6.66	4.36	4.27	8.43	477.4	5.8	4.6	(-2.6-12.5)	0.218
	20-24	Unspecified	14.64	47.15	41.71	52.6	52.07	57.64	53.88	66.91	55.53	83.88	78.91	71.62	389.2	4.9	8.0	(5.4-10.8)	<0.001
	25-34	Food	0.71	0	2.11	1.4	4.87	1.35	1.28	0.61	4.07	1.65	2.05	0.97	36.6	1.4	3.0	(-6.5-13.7)	0.553
	25-34	Medication	0.71	2.12	4.93	7.72	6.96	7.43	6.41	6.66	4.07	6.6	7.68	2.9	308.5	4.1	3.6	(-2.0-9.6)	0.214
	25-34	Unspecified	9.88	28.2	35.21	37.9	43.13	48.65	53.24	44.78	49.42	59.98	52.7	50.27	408.8	5.1	7.3	(5.2-9.5)	<0.001
	35-64	Food	0.26	1.54	0.5	1.23	0.96	0.93	0.23	0.67	0.87	2.13	1.88	1.65	534.6	6.3	9.5	(1.3-18.7)	0.025
	35-64	Medication	4.99	5.15	7.56	9.36	7.91	6.07	8.4	8.66	10.9	8.53	8.37	7.61	52.5	1.5	3.3	(0.4-6.3)	0.025
	35-64	Unspecified	9.45	22.14	25.19	26.84	37.41	38.76	42.01	48.4	52.97	46.07	51.03	58.24	516.3	6.2	10.7	(9.2-12.2)	<0.001
	>=65	Food	0	0	0	0.93	2.7	0	0.85	0	0.79	2.27	1.43	3.43	Inf	Inf	24.8	(6.1-51.4)	0.013
	>=65	Medication	2.06	5.98	11.56	10.24	8.11	15.68	11.84	7.33	15.71	16.61	17.93	15.77	665.5	7.7	9.8	(5.0-15.1)	<0.001
	>=65	Unspecified	9.28	16.96	18.3	25.13	18.02	31.36	16.07	22	29.85	37.75	27.98	32.9	254.5	3.5	7.8	(4.4-11.3)	<0.001
**Females**	**Age (years)**	**All age, all cause**	**14.45**	**36.07**	**44.8**	**54.89**	**55.02**	**53.14**	**60.92**	**62.12**	**68.61**	**75.75**	**82.8**	**84.58**	**485.3**	**5.9**	**9.6**	**(8.9-10.3)**	**<0.001**
	0-4	Food	9.74	1.63	4.91	3.26	4.77	3.02	2.87	5.49	5.33	11.77	5.04	11	12.9	1.1	7.2	(-1.2-16.8)	0.101
	0-4	Medication	0	3.25	3.28	9.78	4.77	3.02	5.74	1.37	5.33	5.23	1.26	2.44	Inf	Inf	-1.2	(-10.8-9.4)	0.812
	0-4	Unspecified	3.25	35.79	27.85	61.93	66.75	54.36	84.69	60.39	87.95	115.07	93.24	84.3	2493.8	25.9	12.4	(9.6-15.4)	<0.001
	5-14	Food	0.75	0.75	0	0	0.74	0.74	0.73	2.85	2.82	2.75	1.34	4.59	512	6.1	25.4	(10.5-44.7)	<0.001
	5-14	Medication	0	0.75	3.74	3.74	2.98	2.21	2.9	2.85	3.52	1.37	1.34	2.63	Inf	Inf	2.3	(-6.5-12.2)	0.619
	5-14	Unspecified	10.47	15.73	28.43	37.42	31.26	44.94	44.97	54.16	46.45	74.2	64.52	62.34	495.4	6	12.8	(10.3-15.4)	<0.001
	15-19	Food	2.91	2.88	1.43	0	2.84	1.38	2.7	4	4	1.33	10.56	6.58	126.1	2.3	15.8	(3.8-30.4)	0.011
	15-19	Medication	1.45	2.88	2.87	12.83	8.53	5.54	8.11	9.34	4	11.96	9.24	7.9	444.8	5.4	7.8	(0.2-16.3)	0.047
	15-19	Unspecified	13.07	25.93	54.51	52.75	59.69	52.59	59.49	85.41	97.28	77.09	87.1	104.02	695.9	8	11.9	(9.1-14.8)	<0.001
	20-24	Food	0	1.51	5.94	2.89	1.4	1.35	3.86	3.69	4.8	1.17	5.71	3.38	Inf	Inf	7.2	(-3.9-20.3)	0.219
	20-24	Medication	3.09	22.71	14.85	18.78	8.4	10.79	11.59	13.52	12.01	10.52	14.86	15.75	409.7	5.1	0.6	(-4.5-6.1)	0.813
	20-24	Unspecified	7.72	31.79	40.1	59.22	63.01	63.41	61.83	66.37	72.06	80.66	94.87	85.51	1007.6	11.1	10.7	(8.0-13.6)	<0.001
	25-34	Food	1.44	2.16	0.72	0	0.72	1.41	0	1.28	1.24	2.94	4.44	4.19	191	2.9	18.1	(6.3-32.6)	0.003
	25-34	Medication	2.15	10.09	9.41	10.14	7.21	7.03	13.49	15.39	15.46	8.83	15.53	16.78	680.5	7.8	8.8	(4.5-13.4)	<0.001
	25-34	Unspecified	6.46	41.08	44.14	40.54	59.13	52.04	64.1	52.57	66.17	66.53	78.19	79.16	1125.4	12.3	9.6	(7.6-11.6)	<0.001
	35-64	Food	1.07	1.05	1.79	1	1.47	0.72	1.86	0.68	0.44	3.25	2.55	2.3	115	2.1	8.5	(1.6-16.0)	0.017
	35-64	Medication	3.47	9.16	13.08	15.57	15.7	11.45	14.38	11.76	14.61	14.28	12.97	21.77	527.4	6.3	6.2	(3.9-8.6)	<0.001
	35-64	Unspecified	11.73	24.07	29.49	39.67	36.56	37.46	38.28	45.91	48.71	47.82	61.9	56.52	381.8	4.8	9.5	(8.1-11.0)	<0.001
	>=65	Food	1.68	0.82	0	0.78	1.53	1.48	2.17	0	1.35	0.65	1.25	3.02	79.8	1.8	7.2	(-5.4-22.4)	0.288
	>=65	Medication	2.53	9.87	18.47	14.89	19.13	13.35	20.95	10.48	8.79	20.84	20.03	15.7	520.6	6.2	4.7	(1.0-8.7)	0.014
	>=65	Unspecified	10.94	19.73	16.86	21.15	16.07	24.48	18.06	22.36	26.36	34.52	35.05	39.25	258.8	3.6	9.8	(6.6-13.1)	<0.001

* Event rates exclude transfers and thus represent separate anaphylaxis events. Individuals may have more than one event within and/or across time periods.

HMDC: Hospital Morbidity Data Collection
EDDC: Emergency Department Data Collection
