# Supplementary material for: Increasing anaphylaxis events in Western Australia identified using four linked administrative datasets
Source: World Allergy Organ J. 2020 Nov 13;13(11):100480. doi: 10.1016/j.waojou.2020.100480 (PMC7677753; doi:10.1016/j.waojou.2020.100480)
Supplement: Multimedia component 4 [file mmc4.pdf]

Table 3. Anaphylaxis event rates , with percentage and fold change 2002-2013, by dataset, by year

		Number of anaphylaxis events per 100,000 persons by year*												Change				
**Category**		**2002**	**2003**	**2004**	**2005**	**2006**	**2007**	**2008**	**2009**	**2010**	**2011**	**2012**	**2013**	**% change 2002-2013**	**fold-change 2002-2013**	**Average annual % increase across all years 2002-2013**	**95% CI**	**p-value for the change**
**Dataset**	**Cause**																	
HMDC	All	11.3	15.21	16.72	20.44	25.85	24.74	25.92	31.96	38.2	41.94	42.61	43.16	281.9	3.8	11.8	(11.0-12.6)	<0.001
	Food	2.64	3.58	4.14	5.92	7.75	7.93	8.98	11.2	14.1	16.49	15.91	16.13	511	6.1	16.3	(14.9-17.8)	<0.001
	Medication	2.96	3.53	5.1	5.82	6.19	5.32	6.45	6.47	8.6	9.18	9.52	10.55	256.4	3.6	10.2	(8.7-11.8)	<0.001
	Unspecified	5.44	7.94	7.17	8.2	11.61	11.21	10.04	13.61	15.02	15.98	16.81	16.21	198	3	9.5	(8.3-10.7)	<0.001
EDDC	All	4.82	25.04	31.32	43.31	43.74	44.54	47.15	51.16	51.81	54.98	55.01	56	1061.8	11.6	9.0	(8.4-9.6)	<0.001
	Food	0	0	0	0.05	0	0	0	0	0	0	0	0.52	Not defined	Not defined	167.4	(70.0-423.3)	<0.001
	Medication	0.05	3.99	5.96	8.7	6.68	5.7	6.54	6.61	5.85	5.48	5.69	5.17	10240	103.4	3.0	(1.5-4.6)	<0.001
	Unspecified	4.61	20.94	25.26	34.56	36.97	38.7	40.52	44.41	45.83	49.46	49.28	50.22	989.4	10.9	9.9	(9.3-10.6)	<0.001
Ambulance	Unspecified	3.37	6.15	6.97	8.1	8.1	7.83	7.05	10.45	15.06	19.16	20.85	21.62	541.5	6.4	16.4	(15.1-17.7)	<0.001
Death	All	0.05	0.1	0.05	0	0.1	0.28	0.09	0.04	0.09	0.17	0.12	0.04	-20	0.8	3.2	(-7.9-16.0)	0.594
	Food	0	0	0	0	0	0	0	0	0	0.04	0.04	0	Not defined	1	61.0	(-6.0-387.7)	0.206
	Medication	0	0	0	0	0	0	0	0	0	0.04	0	0	Not defined	1	43.4	(-25.4-502.2)	0.411
	Unspecified	0.05	0.1	0.05	0	0.1	0.28	0.09	0.04	0.09	0.08	0.08	0.04	-20	0.8	-0.5	(-11.9-12.4)	0.931

* Event rates are dataset specific and thus the same anaphylaxis event may be represented across more than one dataset in a given period. Individuals may have more than one event within and/or across time periods.

HMDC: Hospital Morbidity Data Collection
EDDC: Emergency Department Data Collection
